# A Nutritional and Environmental Analysis of Local Food Pantries Accessible to College Students in Rural North Carolina

**DOI:** 10.13023/jah.0202.03

**Published:** 2020-04-15

**Authors:** Emily E. Frymark, Jonathon L. Stickford, Alisha R. Farris

**Affiliations:** Appalachian State University; Appalachian State University; Appalachian State University

**Keywords:** Appalachia, food pantry, food insecurity, nutrient analysis

## Abstract

**Introduction:**

Food insecurity is a growing concern among college students and is especially prevalent in rural areas. Food pantries often serve as a resource to food insecure individuals yet, their policies, standards, and nutritional quality vary due to the unpredictability of food donations.

**Purpose:**

To examine the nutritional quality of food items and adherence of best practices at local food pantries accessible to college students near a university in rural Appalachia.

**Methods:**

Three food pantries in North Carolina were selected due to their proximity to a local, rural university. Food items were analyzed for nutrient and food group content and compared to national recommended standards for a moderately active 20-year-old male student. Food pantry environments were analyzed using the Healthy Food Pantry Assessment Tool (HFPAT).

**Results:**

All pantries scored in acceptable ranges (39, 59, and 60) on the HFPAT. Food pantries provided 38% of total daily calories and below recommended daily levels for vitamin C (27%), vitamin D (5%), potassium (29%), and calcium (38%), but above recommended levels for sugar (220%), and trans-fat (342%). When all the food from food pantries were combined, they still did not meet food group recommendations, providing: 25% fruit, 50% vegetable, 9% grain, 15% protein, and 20% dairy servings over a 14-day period.

**Implications:**

In general, students who rely on food pantries as their sole source of food do not reach recommend levels for nutrients or food groups. Interventions, programs, and/or policies which increase the healthfulness of food pantry items are warranted to improve the quality of food available to food insecure college students.

## INTRODUCTION

Food insecurity is defined as insufficient food quality or quantity due to lack of financial resources.^1^ It is a public health concern across the U.S. and is widely becoming more recognized among university and college populations. Though national prevalence is unknown, a recent report estimated that 30%–50% of all college students are food insecure.^2^ Further, in a recent study within the rural Appalachian region, over 46% of college students experienced food insecurity, placing rural campuses at the upper end of vulnerability.^3^

Food insecurity can contribute to serious health, social, and academic consequences. Studies involving food insecure individuals have shown associations with diabetes, obesity, hypertension, poor mental health and lower self-rated health.^1^ Among college students specifically, when compared with food secure students, food insecure students are more likely to have a greater body mass index, experience increased stress, anxiety, and depression, consume less decreased fruits and vegetables, and demonstrate poorer academic success.^3^,^4^ Fifty-five percent of students in Students Against Hunger, reported that food insecurity caused them to not buy a required textbook, 53% reported missing a class, and 25% reported dropping a class.^5^

One avenue for combatting food insecurity in communities is through the use of hunger relief programs and organizations, such as food pantries. Food pantries typically provide foods at no-to-little cost and are often distributed through self-selection of a limited number of items, or through a pre-prepared box containing specific items based on availability.^6^ Most pantries are provided with foods from the United States Department of Agriculture’s (USDA) The Emergency Food Assistance Program (TEFAP), as well as donations from nonprofit organizations, local businesses, and members of the community.^7^ Increasingly, many colleges and universities are establishing on-campus food pantries^5^ with the intent to provide a more direct source of assistance to students.

While most food assistance programs in the U.S. have tightly-regulated nutritional standards, the content and composition of foods at food pantries are largely unregulated due to the unpredictability of foods and beverages available, and seasonal variation.^7^ Additionally, meeting the nutritional needs of food pantry patrons can be especially challenging due to these variances and the nutritional quality of food provided.^6^ Previous studies have reported food pantry items to typically be energy-dense, with an abundance of low-nutrient food options^5^ such as, pancake mix, instant macaroni and cheese, and instant mashed potatoes. To the author’s knowledge, no studies have evaluated the quality of food pantry items available to U.S. college students. This study aimed to examine the nutritional quality of foods available and adherence of best practices at food pantries accessible to college students near a university in rural Appalachia. It was hypothesized that food pantry environments would meet acceptable standards, but the food provided would not meet the nutritional needs of college students.

## METHODS

### Setting

Three food pantries within a single county in rural northwest North Carolina were selected for evaluation during April 2018. The three pantries were selected based on their proximity and accessibility to the student population. One pantry was located on the university campus and the other two were within 3 miles of the university and accessible by university bus service. Off-campus pantries which were not supported with governmental funds were excluded. All pantries received food items from community members, local businesses, and nonprofit organizations. One pantry is client choice, while the other two provided the patrons a pre-established box. According to the U.S. Census Bureau, the county had a population of 55,945 people, composed of predominantly white (94.9%) individuals, and the prevalence of poverty was 24.3%.^8^ The university had 18,811 students enroll in the fall academic semester in 2017, with 81.9% white individuals.^9^ Due to the observational nature of the study, no approval by the Institutional Review Board was required.

### Food Pantry Best Practices Measure

The healthfulness of the food pantry environment was assessed using the Healthy Food Pantry Assessment Tool (HFPAT).^10^ The HFPAT is a validated observational survey tool created and piloted by Regional Nutrition Education and Obesity Prevention Centers of Excellence at Washington State University Extension. The HFPAT has been used to measure the food pantry environment as it compares to best practices in food assistance agencies. The tool provides a numeric score on a scale of 0–100. The closer the score is to 100, the more aligned the food pantry environment is to current and healthy best practices. The tool has six main sections: (1) pantry location and entrance; (2) food availability (fresh, canned, frozen); (3) pantry policies; (4) food safety and storage; (5) services for patrons; and (6) other supplementary programs available at the pantry. For scoring, 0, 1, 2, or 3 points were given to the pantry depending on the responses to the questions in the tool. For example, an answer could range from “none available” (0 points) to “wide variety, 7+ types” (3 points). All points were tallied for a final assessment score. The HFPAT was completed at each of the pantry sites within a 2-week period by the same researcher. Tours of the pantries were provided by pantry staff who were available to answer questions if needed. The scores from the HFPAT were used to evaluate food pantry environment and adherence to best practices. Each food pantry was assessed for inventory, and measures were analyzed using descriptive statistics.

### Food Pantry Nutritional Measures

Pantry inventory was analyzed for nutritional content based on the maximum amount of food that could be provided by the pantries to an individual over a 14-day period. This period was chosen because individuals could receive food once every 14 days from two of the three pantries included in the study due to one pantry being client choice, and the other two providing patrons with a pre-established box of food items. In an effort to make nutritional data comparable across pantries, it was assumed that patrons would take one of each item available at the food pantry. All inventory was recorded and nutrient content analyzed (Food Processor Nutrition Analysis Software version 10.12, ESHA, Salem OR) for the macro- and micro-nutrients that are commonly under consumed or are required by the Nutrition Facts food labels.^11^ Commonly under-consumed nutrients include: dietary fiber, potassium, calcium, iron, vitamin A, vitamin D, and vitamin C.^11^ Nutrients required on the Nutrition Facts food label are total calories, total fat, saturated fat, trans fat, total sugar, protein, and sodium. Folate is a nutrient of concern for this age group; therefore, it was also analyzed.^11^ If an item was unavailable in the Food Processor database, a U.S. Department of Agriculture reference item was used. Nutrient content was compared to the dietary reference intake (DRI) recommendations for a moderately active male aged 20 years.

Finally, photos of all pantry food items were taken. This allowed items to be documented based on the food group, the quantity provided, and the serving size per item. Items were categorized into one of ten food groups (fruit, vegetable, grain, plant-based protein, meat, dairy, snack, ready-prepared, dessert, cooking ingredient). For each pantry, servings provided from each food group were compared with USDA daily recommended amounts^11^ for a moderately active male aged 20 years. Whole food items were transposed into cups and ounces using MyPlate standard serving sizes for various food groups. For packaged food items, serving sizes were based on the Nutrition Facts label. The sum of food groups was calculated across the three pantries to estimate the percent recommendation a college student could receive if they collected food boxes from each pantry site. Since pantry items provided food items for a 14-day period, nutrient and food group values were divided by fourteen to reflect daily intake values. Items that were typically used during food preparation but not consumed by themselves (i.e., lemons, seasonings) were excluded from the analysis.

## RESULTS

### Food Pantry Best Practices Measure

Using the HFPAT, Food Pantries 1, 2, and 3 scored 39, 59, and 60 points, respectively. Pantry 1 scored the lowest in the “food available to clients” assessment due to lack of fresh and frozen produce, dairy, and grain products, with 18 points; Pantry 3 scored the highest with 35 points out of 57 total points. Pantry 2 scored the highest in the “frozen, chilled, dry storage, and food safety” section, scoring 8 out of 10 total points because of their clear food safety signage, thermometers, and cleanliness. Pantry 3 also scored the highest in the “services for clients” section, with 6 out of 6 points due to their nutrition education, food demonstrations, and food assistance referral services.

### Nutritional Profile of Pantry Food Items

In total, 159 number of foods were analyzed from the combined pantries. Over the 14-day period assessed, pantries 1, 2, and 3, were capable of distributing 62, 49, and 48 food items, respectively, to an individual (Table 1). When compared with the nutritional recommendations for a moderately active male (20 years) over a 14-day period, food pantries on average provided 38% of total calories and were below recommended levels for vitamin C (27%), vitamin D (5%), potassium (29%), and calcium (38%), but above recommended levels for sugar (220%), and trans-fat (342%). Saturated fat, protein, folate, sodium, and iron all met the recommended DRI. The total sum provided from all three pantries also did not meet recommendations for all nutrients. Vitamin C and potassium only met 82% and 87%, respectively, of nutritional needs (Table 1). Most other nutrients met the DRI, meaning a combination of all the food from the three pantries combined did meet the macro- and micro-nutrient needs of an active male aged 20 years for a 14-day period.

### Food Groups Provided by Pantry Food Items

When all items from all pantry sites were combined, a total of 159 food items, it did not meet 100% of any food group recommendation. The largest food group provided by pantries was vegetables, and the least common food group was grains (Table 2). Vegetables and ready-prepared items were the most dominant pantry items available. Vegetables were mostly available as canned products. When dairy products were available, it was typically in the form of dried milk. Protein would meet 15% of recommendation per day, which is a combination of animal and vegetarian protein products. The most servings per day would come from ready-prepared items, which would be 2.4 servings per day.

## IMPLICATIONS

To the author’s knowledge, this is the first study to examine the nutritional quality of pantry food items and adherence of best practices by pantries accessible to college students. In general, the food pantries did not provide sufficient food groups to meet daily recommendations. While food pantries are intended as a supplemental food supply, many low-income individuals are dependent upon supplemental programs for all of their food needs.^8^ Furthermore, many college students are either not eligible or unaware of eligibility to participate in other food programs such as the Special Supplemental Program for Women, Infants, and Children (WIC) or the Supplemental Nutrition Assistance Program (SNAP), meaning food pantries potentially could be their only source of supplemental food.

A typical range of scores for food pantries, when using the HFPAT, is 35–65 on a scale of 0–100^10^. All pantries in this study fell within the typical range expected. Still, improvements can be made to increase scores, specifically in the areas of providing a variety of (1) fresh and frozen fruits and vegetables, (2) low-fat dairy items, and (3) grains. Previous studies have found these food group items to be lacking in many pantries.^5^,^6^ While the current findings highlight notable nutritional concerns, pantries may be unable to meet a nutritionally adequate diet because they are reliant on donated items and supplemented by the government emergency food supply, which often times is seasonally dependent. Food pantries also may be limited in their storage and refrigeration capabilities, which makes donating to and providing dairy products and fresh produce difficult. Strategies are warranted which would improve the storage capacity of food pantries in order to provide more nutrient-dense items.

The average caloric content provided over the 14-day period failed to meet the DRI of a moderately active male student, providing only 38% of the estimated need, but, combining foods from all pantry sites did meet daily needs. However, many of the calories were provided from non-nutrient dense, ready-made food sources providing over two times the amount of recommended sugar, and almost three and a half times the recommended amount of trans-fat. While trans-fats are slowly being eliminated from the food supply, over-consumption of sugar is a common dietary concern among college students,^11^ and campus food environments often contribute to poor food behaviors.^4^ Non-nutrient dense, ready-made foods are convenient and easy to donate, but they are typically not in-line with Dietary Guidelines for Americans (DGA) recommendations.

Many micronutrients were undersupplied, including calcium, vitamin D, vitamin C, fiber, and potassium. All of these nutrients, with the exception of vitamin C, have been identified as sources of nutritional concern among Americans due to low consumption of dairy, fruits, and vegetables.^11^ Thus, the current findings suggest that pantry foods do little to abate, and may even contribute to, the poor food behavior patterns typically observed among college students, such as unhealthy snacking and the consumption of convenience high-calorie food.^12^ Moreover, over time these patterns increase the risks of high blood pressure, inflammation, weight gain, diabetes, fatty liver disease, and heart disease later in life.^1^

The DGA recommends two cups of fruit and 2–3 cups of vegetables per day for optimal health,^11^ which was not available from the observed food pantries. Food insecure students are more likely to report lower fruit and vegetable consumption,^4^ even though fruits and vegetables are vital in meeting nutrient needs and supporting overall health.^11^ Studies suggest that pantry patrons prefer fresh fruits and vegetables over canned versions and more nutrient-dense food options in general.^13^ However, canned vegetables are easily donated, cheap, and have a long shelf life. In contrast, fresh fruits and vegetables are challenging for food pantries to supply due to donation unpredictability and seasonality of items. Frozen fruit and vegetable items are also difficult to donate, especially by community members, and difficult to store by college students who may not have access to a freezer. Based on the current study findings (i.e., the inability to meet nutritional DRI), patron preferences, and young adult health trends, more options for providing fresh fruits and vegetables to college students are warranted.

The study has several limitations important to note. Pantries were observed once during the spring season, and the potential daily and monthly changes in food availability are not reflected in the current examination. It seems reasonable to believe that food pantries may have more fruits and vegetables during summer months when fresh produce is more readily available. Yet, it also is important to note that many college students are not on campus during the summer months, which makes the timing of the current examination more applicable to the larger student population. One food pantry utilized a patron-choice model where patrons could choose items from all foods that were available. In an effort to make nutritional data comparable across pantries, it was assumed that patrons would take one of each item available. Lastly, all comparisons were based on the DRI of a moderately active male aged 20 years and are therefore not generalizable to those with different activity status, sex, and/or age.

Despite the limitations, results from the current study provide valuable information on the nutrient content of foods available to college students in the rural Appalachian region where a high prevalence of food insecurity exists. Overall, food insecure college patrons, and any patron who rely on food pantries for their sole source of food are not receiving what is recommended for a healthy diet; even when three pantry food boxes were combined. The food provided was deficient in many micronutrients and contained too much sugar and trans fat. This research agrees with current research that states food insecurity contributes to health conditions, and food pantries are insufficient in providing adequate nutrients.^1^,^6^ Thus, more research should be done to address and eliminate food insecurity, especially among college students, and improve the nutritional food content provided from hunger relief programs.

These results could be used to improve the healthfulness of food pantries. Food donation drives should focus on emphasizing unsweetened canned, or fresh fruits and vegetables, plant-based proteins, and whole-grains. Programs and interventions are needed which assist students in budgeting to buy healthier food options that encompass every food group and help educate patrons on the healthy options available at the food pantry. Finally, food pantries and their patrons might benefit from policy change at the federal level, requiring regulations on the type and quality of food provided. Foods currently being donated from the USDA, nonprofits, local businesses, and the community are not meeting nutritional needs and contributing to inappropriate nutrition in this vulnerable population.

**SUMMARY BOX**

**What is already known about this topic?** Food insecurity has increasingly become a public health concern for the college student population. One avenue of combatting food insecurity is through the use of hunger relief organizations, such as food pantries.

**What is added by this report?** This study aimed at examining the nutritional quality of foods available and adherence to best practices at food pantries accessible to college students near a university in rural Appalachia.

**What are the implications for public health practice, policy, and research?** The results of this study can be used to improve the healthfulness of the pantries, educate students on healthy food choices, and have an impact on future policy change for the emergency food supply.

## Figures and Tables

**Table 1:** Daily Nutrients provided per Pantry over a 14-day period Compared to Dietary Reference Intakes

Nutrients (Recommended DRI per day)*	Pantry 1 (n = 62)	Pantry 2 (n = 49)	Pantry 3 (n = 48)	Total Nutrients Provided by All Pantries per day (% of the Recommendation)	Mean Nutrients Provided Across Pantries per day (% of the Recommendation)
Calories (kcal) (2800 kcal)	1090.1	1329.6	775.4	3195.2 (114.1%)	1065.1 (38.0%)
Total Fat (g) (120 g)	17.4	41.0	15.3	73.8 (61.5%)	24.6 (20.5%)
Saturated Fat (g) (40 g)	5.2	16.8	4.4	26.4 (66.1%)	8.8 (22.0%)
Trans Fat (g) (0 g)	1.2	6.7	2.3	10.3 (1028.0%)	3.4 (342.0%)
Fiber (g) (38 g)	21.7	17.2	9.8	48.6 (127.9%)	16.2 (42.6%)
Sugar (g) (25 g)	46.6	67.8	51.1	165.4 (661.5%)	55.1 (220.4%)
Protein (g) (56 g)	45.4	44.1	35.5	125.0 (223.2%)	41.7 (74.5%)
Vitamin A (mcg) (900 mcg)	1645.6	1695.6	248.7	3589.9 (398.9%)	1196.6 (133.0%)
Vitamin C (mg) (90 mg)	21.4	38.3	13.6	73.3 (81.5%)	24.5 (27.2%)
Vitamin D (IU) (600 IU)	26.0	52.9	17.0	95.9 (106.6%)	32.0 (5.3%)
Folate (mcg) (400 mcg)	405.4	373.3	178.6	957.3 (239.3%)	319.1 (79.8%)
Sodium (mg) (2300 mg)	2296.9	2242.4	1345.3	5884.6 (255.9%)	1961.5 (85.3%)
Potassium (mg) (4700 mg)	1433.0	1721.9	918.6	4073.5 (86.7%)	1357.8 (28.9%)
Iron (mg) (8 mg)	14.8	10.3	6.7	31.8 (397.6%)	10.6 (132.5%)
Calcium (mg) (1000 mg)	383.6	399.4	369.9	1152.8 (115.3%)	384.3 (38.4%)

* DRI (Dietary Reference Intake) recommendations are based on a moderately active male aged 20 years.

**Table 2:** Daily Food Groups Provided per Food Pantry over a 14-day period

Food Group (USDA recommended servings per day) *	Pantry 1 Daily amount provided over 14 days	Pantry 2 Daily amount provided over 14 days	Pantry 3 Daily amount provided over 14 days	Sum daily amount provided over 14 days (% of daily recommendation met)
Fruits (2 cups)	0.2 cups	0.2 cups	0.1 cups	0.5 cups (25%)
Vegetables (3 cups)	0.3 cups	0.6 cups	0.6 cups	1.5 cups (50%)
Grain products (8-ounce equivalents)	0.3-ounce equivalents	0.2-ounce equivalents	0.2-ounce equivalents	0.7-ounce equivalents (9%)
Protein (6-ounce equivalents)	0.4-ounce equivalents	0.2-ounce equivalents	0.3-ounce equivalents	0.9-ounce equivalents (15%)
Dairy (3 cups)	0.0 cups	0.5 cups	0.1 cups	0.6 cups (20%)
Snack†	0.1 servings	0.2 servings	0.3 servings	0.6 servings
Dessert†	0.1 servings	0.5 servings	0.6 servings	1.2 servings
Ready-Prepared†	1.4 servings	0.6 servings	0.4 servings	2.4 servings

* United States Department of Agriculture recommendations for a moderately active male aged 20 years.

† Servings based on Nutrition Facts Label
